# Regularised Diffusion–Shock Inpainting

**DOI:** 10.1007/s10851-024-01175-0

**Published:** 2024-04-01

**Authors:** Kristina Schaefer, Joachim Weickert

**Affiliations:** Mathematical Image Analysis Group, Department of Mathematics and Computer Science, Saarland University, E1.7, 66041 Saarbrücken, Germany

**Keywords:** Shock filters, Inpainting, Diffusion, Mathematical morphology, Image processing

## Abstract

We introduce regularised diffusion–shock (RDS) inpainting as a modification of diffusion–shock inpainting from our SSVM 2023 conference paper. RDS inpainting combines two carefully chosen components: homogeneous diffusion and coherence-enhancing shock filtering. It benefits from the complementary synergy of its building blocks: The shock term propagates edge data with perfect sharpness and directional accuracy over large distances due to its high degree of anisotropy. Homogeneous diffusion fills large areas efficiently. The second order equation underlying RDS inpainting inherits a maximum–minimum principle from its components, which is also fulfilled in the discrete case, in contrast to competing anisotropic methods. The regularisation addresses the largest drawback of the original model: It allows a drastic reduction in model parameters without any loss in quality. Furthermore, we extend RDS inpainting to vector-valued data. Our experiments show a performance that is comparable to or better than many inpainting methods based on partial differential equations and related integrodifferential models, including anisotropic processes of second or fourth order.

## Introduction

Image inpainting [17, 32] is the task of filling in missing regions in an image. There are many approaches for solving this task, but in this work we focus on inpainting based on partial differential equations (PDEs). This class of methods is particularly successful in applications with very sparse data such as image compression [20, 28, 42].

PDE-based inpainting methods are often inspired by physical processes. For instance homogeneous diffusion [26, 27, 54] is inspired by heat propagation, and Euler’s elastica inpainting [32, 33] is connected to the elasticity of solids.

Creating a high quality inpainting result with PDE-based methods has some particular challenges. Many operators struggle to bridge large gaps, introduce dissipativity into high contrast images (such as binary ones), or do not reproduce the direction of structures accurately. It is often assumed that high order PDEs such as Euler’s elastica [32, 33] or Cahn–Hilliard inpainting [4] are necessary to address these challenges. However, edge-enhancing diffusion (EED) [49] as a second order integrodifferential process has been shown to provide the desired properties in practice as well [42].

One useful property in the context of inpainting is the fulfilment of a maximum–minimum principle which guarantees that no over- and undershoot are introduced. Most higher order methods violate this principle. EED satisfies a maximum–minimum principle in the continuous case, but to date there is no discretisation with reasonably small stencils available that inherits this property.

**Fig. 1:**
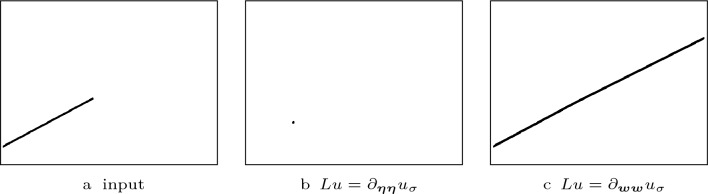
Visual comparison of the steady states of shock filters with different guidance terms with $F(u)= {{\,\textrm{sgn}\,}}(u)$

**Fig. 2:**
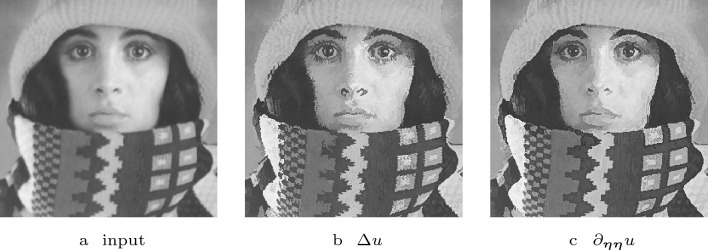
Alteration of an edge-like structure by different shock filters with presmoothing. The steady states of the different shock filters with $F(u)= {{\,\textrm{sgn}\,}}(u)$, $\sigma = 2$ and $\rho = 5$ are shown

*Contributions* In order to address these challenges, we have proposed diffusion–shock inpainting in our conference publication [41]. It is a PDE-based inpainting operator that fulfils the desired properties in practice, while also providing a maximum–minimum principle in the discrete case. This is achieved by combining two time-proven methods: homogeneous diffusion [26, 27, 54] and coherence-enhancing shock filtering [52]. Originally designed with the goal of deblurring, shock filters create sharp edges at the boundary between influence zones of maxima and minima by using the sign of a second derivative operator [30, 35]. However, the coherence-enhancing shock filter can also propagate image structures over large distances without directional or dissipative artefacts, which can be seen in Fig. 1c. In diffusion–shock inpainting, the shock filter propagates edges of image structures without introducing dissipative artefacts. From the newly created structures the homogeneous diffusion term fills in larger homogeneous areas. The synergy of these two methods allows high quality results. Even for high contrast images it reconstructs edges with perfect sharpness and high directional accuracy. Our numerical algorithm satisfies a maximum–minimum principle, and it is optimised for rotation invariance. The experiments in [41] show that diffusion–shock inpainting produces results that rival the quality of state-of-the-art PDE-based inpainting methods such as EED and Euler’s elastica.

In addition to our original conference publication [41], we make the following novel contributions in this work:

1. We introduce regularised diffusion–shock (RDS) inpainting as a regularised version of diffusion–shock inpainting. To this end we replace the ${{\,\textrm{sgn}\,}}$ function that acted as guidance for our original diffusion–shock inpainting model by a sigmoid-like function. This stabilises the process w.r.t. the parameter choice, which allows us to establish a parameter coupling without loss of quality. Thereby we reduce the number of parameters to two, which makes the model more accessible in practice.
2. We give a more detailed description of the numerics.
3. We compare the performance of RDS inpainting with many related approaches. This systematic evaluation reveals that the coherence-enhancing shock term is crucial to the success of RDS inpainting.
4. Finally, we extend our model to vector-valued data which allows the application to colour images.

*Related Work* With the goal of image deblurring, Kramer and Bruckner [30] have proposed a first discrete model of a shock filter already in 1975. Later Osher and Rudin [35] have formulated a first PDE-based approach and coined the term shock filter. Shock filters typically utilise a second derivative operator to identify the influence zones of maxima and minima. Osher and Rudin [35] have considered the Laplacian as well as the second derivative in gradient direction. Alvarez and Mazorra [2] have introduced presmoothing to the second derivative operator in order to robustify the process against noise. As another strategy, Diop and Angulo [13] propose to locally adapt the shock filter to the image to reduce the sensitivity to noise. The coherence-enhancing shock filter of Weickert [52] relies on the second directional derivative in the dominant eigendirection of the structure tensor [18]. In the next section, we will cover the shock filters that are relevant for this paper in more detail. While theoretical results for continuous shock filters are rare, Welk et al. [57] have established well-posedness of 1-D space-discrete and fully discrete shock filters.

Inspired by the implicit presence of shock terms within nonlinear evolutions such as Perona–Malik diffusion [37], self–snakes [40] or the PDE-based version of the Kuwahara–Nagao operator [48], many explicit combinations have been proposed. Typically the shock term of Alvarez and Mazorra [2] is combined with homogeneous diffusion [26, 27, 54], e.g [19, 29], or mean curvature motion [6], e.g [2, 29, 58]. Gilboa et al. [22] rely on complex diffusion. Usually these combinations are used in the context of image enhancement, but not in image inpainting.

RDS inpainting is one of the rare examples of hyperbolic PDEs in inpainting. Another exception is the method of Bornemann and März [5], which was extended by März in [31]. It relies on transport processes that are guided by structure tensor information. Therefore, it is close in spirit to RDS inpainting. However, their paper follows a more algorithmic approach without specifying a compact evolution equation. In our experiments we will compare against this method. Another approach that relies on a hyperbolic concept is the recent inpainting model of Novak and Reinić [34]. It combines a shock filter with the fourth order Cahn–Hilliard PDE. RDS inpaiting is conceptually simpler, as it already achieves the desired filling-in effect with a second order homogeneous diffusion PDE.

To evaluate the performance of RDS inpainting, we compare it to various other PDE-based inpainting operators in our experiments. This includes linear and isotropic processes such as homogeneous diffusion [8, 26, 27, 54] and biharmonic interpolation [14] as its fourth order counterpart. We also consider nonlinear isotropic processes such as total variation (TV) inpainting [44], which can be interpreted as a limiting case of Perona–Malik [37] inpainting with a scalar-valued Charbonnier diffusivity [11]. Moreover, we compare our model to anisotropic approaches such as Tschumperlé’s model [47], which relies on a tensor-driven equation that uses the curvature of integral curves, and to edge-enhancing diffusion [49], which is the core of the state-of-the-art-image compression codec R-EED [42]. Furthermore, we consider the popular higher order inpainting method based on Euler’s elastica [32, 33].

Deep learning techniques have gained popularity for solving inpainting tasks in the past decade [36, 59]. Especially the recent approaches based on probabilistic diffusion [25, 38, 45] have sparked a public discussion due to their highly realistic image generation capabilities. While such models can work well in practice, they typically involve a huge number of parameters that make it very difficult to gain a deeper understanding of their inner workings. Furthermore, they usually do not provide any formal guarantees. On the other hand, our RDS inpainting is a PDE-based model that relies on time-proven components that are carefully selected for the task of image inpainting. The corresponding numerics relies on schemes that are well understood, and it satisfies a maximum–minimum principle. Comparing these two opposite ideologies would not do justice to either of them. Therefore, we do not compare our method to purely learning-based approaches. However, neural networks may also incorporate model-based ideas [23]. This can be used for the implementation of numerically challenging models; e.g. [43] uses a neural network for solving Euler’s elastica for image inpainting. In our experiments, we compare our RDS inpainting to this hybrid approach.

*Organisation of the Paper* In Sect. 2, we review the concept of shock filters. Section 3 introduces the RDS inpainting model in the continuous setting. A numerical scheme with high rotation invariance and stability guarantees in the maximum norm is discussed in Sect. 4. We evaluate our model experimentally in Sect. 5, before concluding the paper in Sect. 6.

## Review of Shock Filters

Shock filters have been introduced with the goal of image sharpening and deblurring. By propagating the values of extrema to their influence zones, shocks are formed at the boundary of these zones. The various ways of characterising these influence zones create different shock filter models that we briefly review in this section.

### PDE-based Morphology

For brightening and darkening of image regions, shock filters rely on the building blocks of mathematical morphology [46]: dilation and erosion. The dilation $\oplus$ of a grey value image $f:\Omega \subset \mathbb {R}^2\rightarrow \mathbb {R}$ replaces the image value in a location $\varvec{x}$ by its supremum within a neighbourhood *B*, the so-called structuring element.^1^ The erosion $\ominus$ uses the infimum instead. The operations are defined as

1 $$\begin{aligned} (f\oplus B)(\varvec{x}) \;&= \; \sup \{ f(\varvec{x} - \varvec{y}) \, | \, \varvec{y} \in B\}, \end{aligned}$$

2 $$\begin{aligned} (f\ominus B)(\varvec{x})\;&=\; \inf \;\{ f(\varvec{x} + \varvec{y})\, | \, \varvec{y} \in B\}. \end{aligned}$$

For shock filters, their PDE-based formulations are more popular. Dilation/erosion *u* with a disk-shaped neighbourhood of radius *t* correspond to the solution $u(\varvec{x},t)$ of

3 $$\begin{aligned} \partial _t u \; = \; \pm \, |\varvec{\nabla }u| \end{aligned}$$

with the initial image $u(\varvec{x}, 0) = f(x)$ and reflecting boundaries [1, 3, 7]. The $+$ sign corresponds to dilation, and − yields erosion. We denote the spatial nabla operator by $\varvec{\nabla }= (\partial _x, \partial _y)^\top$, and $|\cdot |$ is the Euclidean norm.

### Shock Filters

In order to achieve the desired sharpening, shock filters apply dilation and erosion adaptively: In influence zones of maxima they use dilation, and in influences zones of minima they apply erosion. This switch is modelled by considering the sign of a second derivative operator. In general, shock filters have the form

4 $$\begin{aligned} \partial _t u \; = \; - F(Lu) |\varvec{\nabla }u|\,. \end{aligned}$$

The *guidance term* *F*(*Lu*) determines the behaviour of the shock filter. It consists of the second order derivative operator *Lu* and the *guidance function* $F:\mathbb {R}\rightarrow [-1,1]$, which has to retain the sign of its input.

We distinguish the shock filter types by their second order derivative operator *Lu*. Osher and Rudin [35] considered the Laplacian $Lu =\Delta u$ and the second derivative $Lu=\partial _{\varvec{\eta \eta }} u$ in the normalised gradient direction $\varvec{\eta } \parallel \varvec{\nabla } u$. They argue that $\partial _{\varvec{\eta }\varvec{\eta }} u$ gives better results. This is in accordance with findings of Haralick [24], who favours the zero crossings of $\partial _{\varvec{\eta }\varvec{\eta }} u$ over the ones of $\Delta u$ as edge detectors. We confirm this in Fig. 2. Both filters result in a non-flat, segmentation-like steady state and sharpen the image without drastically changing its structure. However, the second derivative in gradient direction yields cleaner edges.

To robustify the process against noise, Alvarez and Mazorra [2] introduced a presmoothing to the derivative operator and used $Lu = \partial _{\varvec{\eta }\varvec{\eta }} u_\sigma$, where $u_\sigma = K_\sigma * u$ denotes the convolution of the image with a Gaussian of standard deviation $\sigma$. Applying this presmoothing may drastically change the structure of the evolving image.

For his coherence-enhancing shock filter, Weickert [52] uses the second derivative in direction of the dominant eigenvector $\varvec{w}$ (i.e. the eigenvector with the larger corresponding eigenvalue) of the structure tensor $\varvec{J}_\rho (\varvec{\nabla }u) = K_\rho *(\varvec{\nabla }u \varvec{\nabla }u^\top )$ [18]. Hence, the coherence-enhancing shock filter relies on $Lu = \partial _{\varvec{w}\varvec{w}} u_\sigma$. We use $\varvec{J}_\rho (\varvec{\nabla }u_\sigma )$ instead of $\varvec{J}_\rho (\varvec{\nabla }u)$ since it yields better results for our application. Hence, the dominant eigenvector $\varvec{w}$ depends on the noise scale $\sigma$ and the integration scale $\rho$. As is common in the structure tensor literature, we do not make this explicit by adding extra indices. As the dominant eigenvector of the structure tensor corresponds to the direction of the largest local contrast, this filter has a coherence-enhancing effect. Similar observations exist in the context of coherence-enhancing diffusion [51].

While the choice of *Lu* determines the main behaviour of the shock filter, one may also choose from various guidance functions *F*. In our conference publication [41] we relied on the ${{\,\textrm{sgn}\,}}$ function as the most widely used choice. However, different *sigmoid*-like functions have been used in the literature, including $\arctan$ functions [22] or hyperbolic tangent functions [19]. For our RDS inpainting, we rely on *sigmoid*-like functions as a regularised alternative to the ${{\,\textrm{sgn}\,}}$ function. We will evaluate the benefits of this choice in our experiments.

In Fig. 1, we investigate the potential of shock filters to propagate structures over large distances by the example of a partial line. The Alvarez–Mazorra model shrinks the line to a small disk-like shape. The coherence-enhancing shock filter elongates the line perfectly over a distance of more than 200 pixels in a direction that is not grid aligned. Moreover, it creates a perfectly sharp result without introducing any dissipativity. This quality is exceptional for PDE-based methods. Therefore, we choose the coherence-enhancing shock filter as a key component of our RDS inpainting.

## Regularised Diffusion–Shock Inpainting

For image inpainting, we decompose the rectangular image domain $\Omega$ into two regions: The known data locations are represented by the *inpainting mask* $K\subset \Omega$, and the unknown values are located in the *inpainting domain* $\Omega \setminus K$. In the inpainting domain, a PDE-based inpainting method applies a suitable differential operator until the process converges. For RDS inpainting a weighted combination of a regularised coherence-enhancing shock filter and homogeneous diffusion takes that role.

As we show in Fig. 1, the coherence-enhancing shock filter can propagate edge-like structures over arbitrarily large distances with perfect sharpness and directional accuracy. However, the width of the created structures is limited by the presmoothing scale $\sigma$. Here, homogeneous diffusion is the ideal partner: It efficiently fills the missing large areas from the sharp edges created by the shock filter.

In order to achieve this behaviour, we apply a weighted combination of the two components such that the shock term dominates near edges, and the diffusion term takes over in more homogeneous regions. We model this by means of a Charbonnier weight function [11]

5 $$\begin{aligned} g\left( |\varvec{\nabla }u_\nu |^2\right) \; = \; \frac{1}{\sqrt{1+ |\varvec{\nabla }u_\nu |^2/\lambda ^2}} \end{aligned}$$

with the Gaussian-smoothed image $u_\nu = K_\nu * u$. It is a decreasing function with range (0, 1], for which we have $g(0) = 1$ and $g(|\varvec{\nabla }u_\nu |^2) \rightarrow 0$ for $|\varvec{\nabla }u_\nu |^2 \rightarrow \infty$. By presmoothing the image before computing the gradient, we locally average structural information and stabilise the process w.r.t. noise.

With that, our *regularised diffusion–shock (RDS) inpainting* is based on the PDE

6 $$\begin{aligned} \partial _t u= & {} g\left( |\varvec{\nabla }u_\nu |^2\right) \, \Delta u\nonumber \\{} & {} - \Big (1-g\left( | \varvec{\nabla }u_\nu |^2\right) \!\Big ) \, S_\varepsilon \left( \partial _{\varvec{w}\varvec{w}} (u_\sigma ) \right) \, | \varvec{\nabla }u | \,. \end{aligned}$$

We use Dirichlet data at the boundaries $\partial K$ of the inpainting mask and reflecting boundary conditions on the image domain boundary $\partial \Omega$. By $S_\varepsilon$ we denote a sigmoidal function with a regularisation parameter $\varepsilon >0$. This adds additional regularisation to the model: It softens the transition from dilation to erosion in the shock term. This choice is reminiscent of the regularisation of the Chan–Vese model for segmentation [10], which relies on a rescaled family of $\arctan$ functions. In our experiments, we will use

7 $$\begin{aligned} S_\varepsilon (x) \;=\; \frac{2}{\pi } \arctan \left( \frac{x}{\varepsilon }\right) \,. \end{aligned}$$

**Fig. 3:**
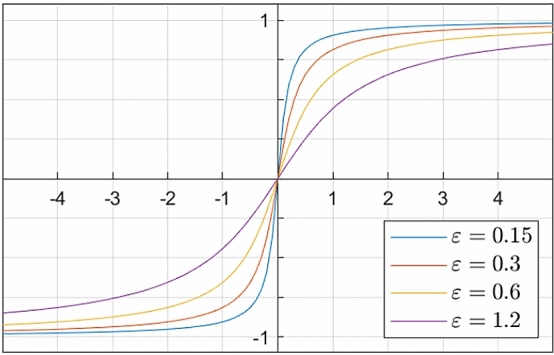
Effect of the regularisation parameter $\varepsilon$ on $S_\varepsilon (x)=\frac{2}{\pi }\arctan \left( \frac{x}{\varepsilon }\right)$

As depicted in Fig. 3 the regularisation parameter $\varepsilon$ determines the steepness of the $\arctan$ function. For $\varepsilon \rightarrow 0$, we arrive at the diffusion–shock inpainting model from our conference publication [41], which uses a ${{\,\textrm{sgn}\,}}$ function instead.

### Parameter Coupling

The model parameters of RDS inpainting fall into two natural categories: The noise scale $\sigma$, the integration scale $\rho$, and the edge scale $\nu$ serve as spatial scale parameters within the image domain, whereas the contrast parameter $\lambda$ and the regularisation parameter $\varepsilon$ are tonal scale parameters acting in the codomain. This classification allows to introduce a parameter coupling that reduces the five parameters to only two. This greatly eases the practical applicability of RDS inpainting.

The noise scale $\sigma$ determines the width of the structures created by the shock term. In the computation of the structure tensor $\varvec{J}_\rho (\varvec{\nabla }u_\sigma )$ it removes noise and small-scale details. In order to avoid cancellation effects of gradients with opposite orientation and very wide borders of edge-like structures $\sigma$ should be chosen relatively small. The integration scale $\rho$ allows averaging of directional information without cancellation effects. It stabilises the directional accuracy of the coherence-enhancing shock filter. A larger $\rho$ usually gives a better directional accuracy. Therefore one should usually choose $\rho > \sigma$. The edge scale $\nu$ of the weighting function averages structure information locally. Especially in the beginning of the evolution, there may not be sufficient unambiguous structural information available for the shock term to identify meaningful structures. Presmoothing the gradient information with a sufficiently large edge scale allows to assign a suitable weighting to the diffusion term and the shock term.

**Fig. 4:**
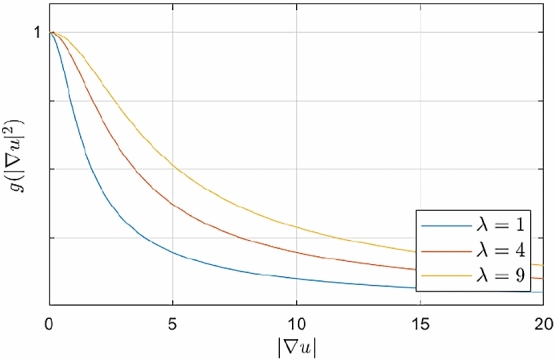
Effect of the contrast parameter $\lambda$ on the Charbonnier diffusivity

The contrast parameter $\lambda$ and the regularisation parameter $\varepsilon$ control the tonal behaviour of RDS inpainting in its codomain. As depicted in Fig. 4, the contrast parameter $\lambda$ determines how fast the Charbonnier weight decreases. A smaller $\lambda$ leads to a larger zone of gradient values in which the shock term dominates. The regularisation parameter $\varepsilon$ softens the transition from dilation to erosion and avoids too rapid edge formation in the beginning. For small values of the second derivative operator in the guidance term, it shrinks the strength of the shock filter. A small $\varepsilon$ yields a very harsh transition, and a large $\varepsilon$ results in a more gradual evolution towards the discontinuous steady state.

In order simplify the parameter optimisation in practice, we calibrate the five parameters by a single one in each category. For that purpose, we couple the spatial scales to each other. We choose $\rho =\nu = 1.6 \cdot \sigma$ since it works well for all of the experiments that we performed. Moreover, we couple the tonal parameters $\lambda$ and $\varepsilon$. In our experiments, we use $\varepsilon = 0.15 \cdot \lambda$.

With that we addressed the main drawback of our conference publication [41]: the large number of parameters. We reduced the number of parameters that have to be optimised to two. This makes RDS inpainting easier to use in practice. Let us also emphasise that the newly introduced regularisation allows the parameter coupling without a loss in quality in comparison to our original diffusion–shock inpainting from [41]. Our experiments demonstrate that RDS inpainting gives better results than the original diffusion–shock inpainting from [41] if parameter coupling is applied to it.

### Extension to Vector-Valued Data

Let us now consider a vector-valued image $f:{\Omega }\rightarrow \mathbb {R}^{n_c}$ with $n_c$ channels. Vector-valued data are common in image processing as they are typically used for RGB colour images or hyperspectral data. Since our RDS inpainting relies on structural information for guidance, a simple channelwise application is not appropriate: The shock term might create shocks at different locations for each channel, and the weighting of the shock term and the diffusion term could vary across the channels.

By utilising a joint squared gradient magnitude [12] as well as a joint structure tensor [21, 50], we can synchronise the operations across all channels. The joint Charbonnier weight is given by

8 $$\begin{aligned} g\left( \frac{1}{n_c}\sum \limits _{c=1}^{n_c}|{{\,\mathrm{\varvec{\nabla }}\,}}(u_c)_\nu |^2\right) \,, \end{aligned}$$

and the joint structure tensor is

9 $$\begin{aligned} \frac{1}{n_c} \sum \limits _{c=1}^{n_c} \varvec{J}_\rho ({{\,\mathrm{\varvec{\nabla }}\,}}(u_c)_\sigma )\,. \end{aligned}$$

This strategy is also in line with the multi-channel version of the coherence-enhancing shock filter from [52].

Overall, for the vector-valued image $\varvec{u}:{\Omega }\times [0,\infty ) \rightarrow \mathbb {R}^{n_c}$ the following evolution describes RDS inpainting in the inpainting domain:

10 $$\begin{aligned} \partial _t u_c \; =&\; g\left( \frac{1}{n_c}\sum \limits _{c=1}^{n_c}|{{\,\mathrm{\varvec{\nabla }}\,}}(u_c)_\nu |^2\right) \, \Delta u_c \nonumber \\ \;&-\; \left( 1-g\left( \frac{1}{n_c}\sum \limits _{c=1}^{n_c} |{{\,\mathrm{\varvec{\nabla }}\,}}(u_c)_\nu |^2\right) \right) \,\nonumber \\&\quad S_\varepsilon \left( \partial _{\varvec{w}\varvec{w}} ((u_c)_\sigma ) \right) \, | \varvec{\nabla }u_c | \end{aligned}$$

for each channel *c*. As in the scalar-valued case, we use Dirichlet data at the boundaries $\partial K$ of the inpainting mask, and reflecting boundary conditions on the image domain boundary $\partial \Omega$.

## Numerical Algorithm

In order to apply our model to a discrete image $(f_{i,j})$ with pixels (*i*, *j*) and grid size *h*, we discretise (6) with an explicit scheme. The discrete evolving image $u^k_{i,j}$ is an approximation of $u(\varvec{x},t)$ in the cell-centred location $\varvec{x}= \left( i-\frac{1}{2}, j-\frac{1}{2}\right) ^\top$ at the time $t=k\tau$, where *k* is the iteration number and $\tau$ is the time step size. For the time derivative, we apply a forward difference

11 $$\begin{aligned} (\partial _t u)_{i,j}^k \;=\; \frac{u_{i,j}^{k+1}-u_{i,j}^k}{\tau }. \end{aligned}$$

The spatial derivatives are evaluated at the old time level *k*.

### Approximation of Homogeneous Diffusion

For the approximation of the homogeneous diffusion term $\Delta u$ we rely on the $\delta$-stencil of Welk and Weickert [56]. They propose a convex combination of axial and diagonal central differences in order to achieve a high degree of rotation invariance. The corresponding stencil is given by12with a weight $\delta \in [0,1]$. As is common in the numerical literature, the stencil notation specifies the discrete convolution weights in the locationsAn explicit discretisation of the homogeneous diffusion equation $\partial _t u = \Delta u$ with this stencil results in the following iterative scheme:

13 $$\begin{aligned} u^{k+1}_{i,j} \;&= \; u_{i,j}^k \left( 1- \frac{4-2\delta }{h^2}\tau \right) \nonumber \\&\quad + \frac{1-\delta }{h^2}\tau \left( u_{i+1,j}^k + u_{i-1,j}^k + u_{i,j+1}^k + u_{i,j-1}^k \right) \nonumber \\&\quad \!+\! \frac{\delta }{2h^2}\tau \left( u_{i+1,j+1}^k \!+\! u_{i+1,j-1}^k \!+\! u_{i-1,j-1}^k \!+\! u_{i-1,j+1}^k \right) \end{aligned}$$

with $u^0_{i,j} = f_{i,j}$. Thus, $u^{k+1}_{i,j}$ is a convex combination of the image data at time level *k*, if

14 $$\begin{aligned} \tau \;\le \; \frac{h^2}{4-2\delta } \;=:\; \tau _D\,. \end{aligned}$$

This implies stability in terms of the maximum–minimum principle

15 $$\begin{aligned} \min _{n,m} f_{n,m} \; \!\le \!\; u^k_{i,j} \;\le \; \max _{n,m} f_{n,m}\; \text{ for } \text{ all }\,i, j,\hbox { and for }k \ge 0. \end{aligned}$$

### Approximation of Dilation and Erosion

To discretise the morphological terms $\pm |\varvec{\nabla }u|$, we rely on upwind schemes. This type of discretisation adaptively selects a one-sided difference that reflect the local transport direction. For dilation and erosion, the classical Rouy–Tourin upwind schemes [39] are a popular choice. However, for the discretisation of the morphological terms $\pm |\varvec{\nabla }u|$ in our RDS inpainting, we follow Welk and Weickert [56] again. They combine the classical axial Rouy–Tourin upwind scheme with its diagonal variant with a weight $\delta$. For the dilation term $|\varvec{\nabla }u|$, the resulting scheme is given by

16 $$\begin{aligned} |\varvec{\nabla }u|_{i,j}^k&= \tfrac{1-\delta }{h} \, \big ( \max \, \lbrace u_{i+1,j}^k \!-\! u_{i, j}^k,\; u_{i-1,j}^k \!-\! u_{i, j}^k,\; 0\rbrace ^2 \nonumber \\&\quad + \max \, \lbrace u_{i,j+1}^k \!-\! u_{i, j}^k,\; u_{i,j-1}^k \!-\! u_{i, j}^k,\;0\rbrace ^2 \big )^\frac{1}{2} \nonumber \\&\quad +\;\tfrac{\delta }{\sqrt{2}h} \, \big ( \max \, \lbrace u_{i+1,j+1}^k - u_{i, j}^k,\; u_{i-1,j-1}^k \!-\! u_{i, j}^k,\; 0\rbrace ^2 \nonumber \\&\quad + \max \, \lbrace u_{i-1,j+1}^k \!-\! u_{i, j}^k,\; u_{i+1,j-1}^k \!-\! u_{i, j}^k,\;0\rbrace ^2 \big )^\frac{1}{2} \end{aligned}$$

with the weight $\delta \in [0,1]$ and $u^0_{i,j} = f_{i,j}$. The Rouy–Tourin upwind schemes are designed to adapt the one-sided differences to the local transport direction. Dilation transports bright values into dark regions, and erosion propagates dark values into bright regions. Hence, they have opposing transport directions. Therefore, upwind schemes for erosion flip the finite differences that are present in the dilation scheme. Here, we rely on the work of Welk and Weickert [56] as well. They propose to discretise the erosion term $-|\varvec{\nabla }u|$ as

17 $$\begin{aligned} -|\varvec{\nabla }u|_{i,j}^k&= -\tfrac{1-\delta }{h} \, \big ( \max \, \lbrace u_{i,j}^k \!-\! u_{i+1, j}^k,\; u_{i,j}^k \!-\! u_{i-1, j}^k,\; 0\rbrace ^2 \nonumber \\&\quad + \max \, \lbrace u_{i,j}^k \!-\! u_{i, j+1}^k ,\; u_{i,j}^k \!-\! u_{i, j-1}^k,\;0\rbrace ^2 \big )^\frac{1}{2} \nonumber \\&\quad -\;\tfrac{\delta }{\sqrt{2}h} \, \big ( \max \, \lbrace u_{i,j}^k \!\quad -\! u_{i+1,j+1}^k,\; u_{i,j}^k \!-\! u_{i-1,j-1}^k,\;0\rbrace ^2 \nonumber \\&\quad + \max \, \lbrace u_{i,j}^k \!-\! u_{i-1,j+1}^k ,\; u_{i,j}^k \!-\! u_{i+1,j-1}^k ,\;0 \rbrace ^2 \big )^\frac{1}{2} \;. \end{aligned}$$

An explicit scheme with forward difference in time and space discretisation (16) or (17) results in the following iterative schemes for dilation (18) and erosion (19):

18 $$\begin{aligned} u^{k+1}_{i,j}&= u^k_{i,j}+ \tfrac{1-\delta }{h}\tau \, \big ( \max \, \lbrace u_{i+1,j}^k \!-\! u_{i, j}^k,\; u_{i-1,j}^k \!-\! u_{i, j}^k,\; 0\rbrace ^2 \nonumber \\&\quad + \max \, \lbrace u_{i,j+1}^k \!-\! u_{i, j}^k,\; u_{i,j-1}^k \!-\! u_{i, j}^k,\;0\rbrace ^2 \big )^\frac{1}{2} \nonumber \\&\quad +\;\tfrac{\delta }{\sqrt{2}h}\tau \, \big ( \max \, \lbrace u_{i+1,j+1}^k \!-\! u_{i, j}^k,\; u_{i-1,j-1}^k \!-\! u_{i, j}^k,\; 0\rbrace ^2\nonumber \\&\quad + \max \, \lbrace u_{i-1,j+1}^k \!-\! u_{i, j}^k,\; u_{i+1,j-1}^k \!-\! u_{i, j}^k,\;0\rbrace ^2 \big )^\frac{1}{2}, \end{aligned}$$

19 $$\begin{aligned} u^{k+1}_{i,j} = u^k_{i,j}&-\tfrac{1-\delta }{h}\tau \, \big ( \max \, \lbrace u_{i,j}^k \!-\! u_{i+1, j}^k,\; u_{i,j}^k \!-\! u_{i-1, j}^k,\; 0\rbrace ^2 \nonumber \\&\quad + \max \, \lbrace u_{i,j}^k \!-\! u_{i, j+1}^k ,\; u_{i,j}^k \!-\! u_{i, j-1}^k,\;0\rbrace ^2 \big )^\frac{1}{2} \nonumber \\&\quad -\;\tfrac{\delta }{\sqrt{2}h} \tau \, \big ( \max \, \lbrace u_{i,j}^k \!-\! u_{i+1,j+1}^k,\; u_{i,j}^k \!-\! u_{i-1,j-1}^k,\;0\rbrace ^2 \nonumber \\&\quad + \max \, \lbrace u_{i,j}^k \!-\! u_{i-1,j+1}^k ,\; u_{i,j}^k \!-\! u_{i+1,j-1}^k ,\;0 \rbrace ^2 \big )^\frac{1}{2} \, , \end{aligned}$$

with $u^0_{i,j} = f_{i,j}$. They satisfy the maximum–minimum principle (15) if

20 $$\begin{aligned} \tau \;\le \; \frac{h}{\sqrt{2}\,(1-\delta ) + \delta } \;=:\; \tau _M. \end{aligned}$$

In order to show this, one has to consider all possible schemes resulting from the different cases of the $\max$ operations. For the sake of brevity, let us now sketch how to show the stability for the dilation process (18) in the following case:

21 $$\begin{aligned} \max \lbrace u_{i+1,j}^k\, , u_{i-1,j}^k \, , u_{i,j+1}^k \, , u_{i,j-1}^k \, , u_{i,j}^k \rbrace \;&=\; u_{i+1,j}^k , \end{aligned}$$

22 $$\begin{aligned} \max \lbrace u_{i+1,j+1}^k\, , u_{i-1,j+1}^k \, , u_{i+1,j-1}^k \, , u_{i-1,j-1}^k \, , u_{i,j}^k \rbrace \;&=\; u_{i+1,j+1}^k \, . \end{aligned}$$

Clearly the statement (15) is fulfilled for $k= 0$ as $u^0_{i,j} = f_{i,j}$. Thus, it is sufficient to show that $\min \limits _{n,m} u^k_{n,m} \le u^{k+1}_{n,m} \le \max \limits _{n,m} u^k_{n,m}$. With (21) and (22), the dilation scheme (18) has the upper bound

23 $$\begin{aligned} u^{k+1}_{i,j} \;&\le \; u_{i,j}^k + \frac{1-\delta }{h}\tau \sqrt{2}\left( u_{i+1,j}^k - u_{i, j}^k\right) \nonumber \\&\quad + \frac{\delta }{h}\tau \left( u_{i+1,j+1}^k - u_{i, j}^k\right) \nonumber \\&= \; u_{i,j}^k \left( 1- \frac{\sqrt{2}(1-\delta ) + \delta }{h} \tau \right) \nonumber \\&\quad + \frac{\delta }{h}\tau u_{i+1,j+1}^k + \frac{1-\delta }{h}\tau \sqrt{2}u_{i+1,j}^k \end{aligned}$$

If (20) holds, this is a convex combination. Therefore, we have

24 $$\begin{aligned} u^{k+1}_{i,j}\; \le \;\max _{n,m} u^k_{n,m}\; \le \;\max _{n,m} f_{n,m} \, . \end{aligned}$$

Moreover, we have

25 $$\begin{aligned} u^{k+1}_{i,j} \; \ge \;\min _{n,m} u^k_{n,m} \;\ge \;\min _{n,m} f_{n,m}. \end{aligned}$$

Thus, the discretisation of dilation (18) satisfies a maximum–minimum principle for the case (21), (22). The other cases work in the same way. The stability of the erosion evolution can be shown analogously.

### Discretisation of RDS Inpainting

To discretise the full RDS inpainting equation, we need to discretise the guidance term of the shock filter and the weight $g(|{{\,\mathrm{\varvec{\nabla }}\,}}u_\nu |^2)$ as well. For that we approximate all first order partial derivatives $\partial _x u$ and $\partial _y u$ in the gradient as well as in the structure tensor with Sobel operators [15], since they offer a good rotation invariance:26The Gaussian convolutions are computed in the spatial domain with a sampled and renormalised Gaussian, which is truncated at five times its standard deviation. We compute the normalised dominant eigenvector $\varvec{w} = (c, s)^\top$ of the structure tensor analytically, since it is a symmetric $2 \times 2$ matrix. For the computation of $\partial _{\varvec{w}\varvec{w}} v$ we use

27 $$\begin{aligned} \left( \partial _{\varvec{w} \varvec{w}} v\right) _{i,j} ^k \;=\; \left( c^2 \, \partial _{xx}v + 2 cs \, \partial _{xy}v + s^2 \, \partial _{yy}v\right) _{i,j}^k \end{aligned}$$

where second order partial derivatives are approximated with the following finite differences:

28 $$\begin{aligned} (\partial _{xx} v)_{i,j}^k \;&\approx \; \frac{v^k_{i+1,j} - 2v^k_{i,j} + v^k_{i-1,j}}{h^2}\, , \end{aligned}$$

29 $$\begin{aligned} (\partial _{yy} v)_{i,j}^k \;&\approx \; \frac{v^k_{i,j+1} - 2v^k_{i,j} + v^k_{i,j-1}}{h^2}\, ,\end{aligned}$$

30 $$\begin{aligned} (\partial _{xy} v)_{i,j}^k \;&\approx \; \frac{v^k_{i+1,j+1} + v^k_{i-1,j-1} -v^k_{i-1,j+1} - v^k_{i+1,j-1}}{4h^2}\,. \end{aligned}$$

We implement reflecting boundary conditions by adding a layer of mirrored dummy pixels around the image borders. For the Gaussian convolution of the first order derivatives within the structure tensor, we enforce this by imposing zero values at the image boundaries.

Putting everything together yields the following explicit scheme for the RDS inpainting evolution (6):

31 $$\begin{aligned} \frac{u^{k+1}_{i,j}- u^k_{i,j}}{\tau }&= g_{i,j}^k \cdot \big (\Delta u\big )_{i,j}^k - \left( 1-g_{i,j}^k\right) \cdot \nonumber \\&\quad S_\varepsilon \left( \left( \partial _{\varvec{w} \varvec{w}} u_\sigma \right) ^k_{i,j}\right) \, |\varvec{\nabla }u|^k_{i,j} \end{aligned}$$

with initial condition $u^0_{i,j} = f_{i,j}$. It inherits its stability from the schemes for diffusion and morphology:

#### Theorem 1

**(Stability of the RDS Inpainting Scheme)**

Let the time step size $\tau$ of the scheme (31) be restricted by

32 $$\begin{aligned} \tau \; \le \; \min \,\{\tau _D, \, \tau _M\} \end{aligned}$$

with $\tau _D$ and $\tau _M$ as in (14) and (20).

Then the scheme satisfies the discrete maximum–minimum principle

33 $$\begin{aligned} \min _{n,m} f_{n,m} \; \le \; u^k_{i,j} \;\le \; \max _{n,m} f_{n,m}\; \qquad \text{ for } \text{ all }\,i, j, \hbox { and for } k \ge 0. \end{aligned}$$

#### Proof

If $\tau \le \min \,\{\tau _D, \, \tau _M\}$, it follows from the stability of the diffusion and morphological processes that

$$\begin{aligned} u^{k+1}_{i,j} \;&=\; u^k_{i,j} +\tau g_{i,j}^k \cdot \big (\Delta u\big )_{i,j}^k - \left( 1-g_{i,j}^k\right) \cdot \\&\quad \,\tau S_\varepsilon \big (\left( \partial _{\varvec{w} \varvec{w}}u_\sigma \right) ^k_{i,j}\big ) \, |\varvec{\nabla }u|^k_{i,j}\\&\le \; g_{i,j}^k \max _{n,m} f_{n,m} + \left( 1-g_{i,j}^k\right) \max _{n,m} f_{n,m} \\ \;&=\;\max _{n,m} f_{n,m} \; . \end{aligned}$$

Analogously, one can show the condition $\, \min \limits _{n,m} f_{n,m} \, \le \, u^k_{i,j}$.

For good rotation invariance, we follow the suggestion of Welk and Weickert [56] and use $\delta =\sqrt{2} - 1$. Thus, for $\,h=1\,$ our scheme satisfies a maximum–minimum principle for $\,\tau \,\le \, \tau _D \,\approx \, 0.31$. This shows a clear advantage of RDS inpainting over EED inpainting [42, 55], for which there is currently no numerical algorithm that fulfils a maximum–minimum principle on a bounded stencil.

In order to use this numerical scheme for vector-valued data, we discretise $\Delta u_c$, $\pm |\varvec{\nabla }u_c|$ and $\partial _t u_c$ for each channel $c \in \{1...n_c\}$ and apply a channel coupling to the weight and structure tensor as indicated by Eq. (10). The stability limit does not change.

## Experiments

### Comparison to Related Approaches

Combinations of smoothing and shock filtering, either explicitly or implicitly, are rare in image inpainting, but fairly common for image enhancement. Many methods combine mean curvature motion (MCM) [6] for smoothing with the shock term of Alvarez and Mazorra [2], see e.g. [2, 40, 58]. These methods are unable to perform inpainting, since MCM is not suitable for inpainting in general [9], and the width of structures propagated by shock filters is limited to the presmoothing scale. Therefore, we compare RDS inpainting with other shock-smoothing combinations that rely on homogeneous diffusion instead. This includes the methods of Kornprobst et al. [29], Fu et al. [19] and Perona–Malik diffusion [37]. Table 1 shows the corresponding evolution equations. To isolate the effect of the shock term, we also include a variant of RDS inpainting that uses the shock term of Alvarez and Mazorra [2] instead of the proposed coherence-enhancing shock term.

**Fig. 5:**
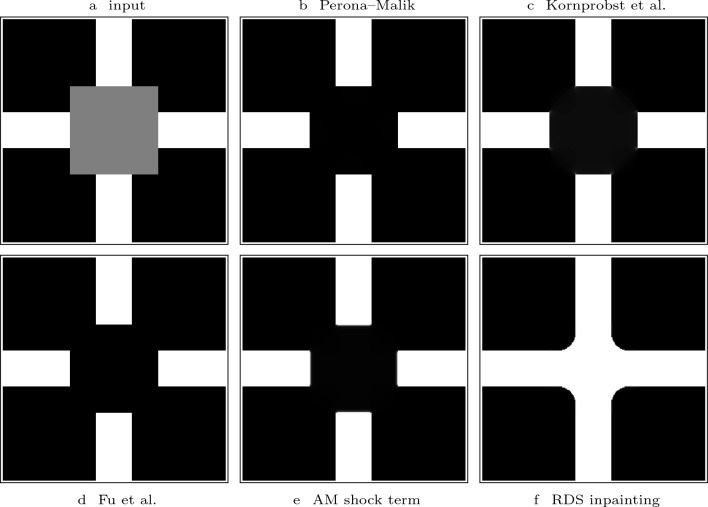
Comparison of RDS inpainting to related approaches. Parameters: **b** $\lambda = 2$. **c** $\beta =\gamma =1$, $\sigma = 1.5$, $T= 30$. **d** $\ell _1 = 1$, $T_1 = 40$, $T_2 = 35$, $\sigma = 2$. **e** RDS inpainting with shock term of Alvarez and Mazorra, $\sigma =2$, $\nu = 4$, $\lambda = 1.5$. **f** $\sigma =2$, $\lambda = 1.5$

The experiment in Fig. 5 shows the result of our comparison. It is inspired by a popular experiment for Cahn–Hilliard inpainting from Fig. 2 in the paper [4] . The goal is the reconstruction of a cross. Clearly, RDS inpainting gives the best result: It reconstructs a binary, cross-like shape. All other methods are unable to connect the white bars. Moreover, RDS inpainting also gives a sharper result than the original Cahn–Hilliard inpainting from Fig. 2 in [4]. The comparison of Fig. 5e, f emphasises that the coherence-enhancing shock term is crucial for the performance of RDS inpainting. Moreover, it should be noted that our RDS inpainting with parameter coupling requires to specify only two parameters, in contrast to the competing explicit combinations: The method by Kornprobst et al. [29] uses four parameters, and the approach of Fu et al. [19] has five different parameters. Thus, our approach is easier to use in practice.

**Table 1:** PDEs or energies corresponding to the inpainting operators that we compare to in our experiments.

Operator	Evolution equation / Energy functional
Hom. Diff. [26, 27, 54]	$\partial _t u \;=\; \Delta u$
Biharm. Interpol. [14]	$\partial _t u \;=\; -\Delta ^2 u$
Perona–Malik [37]	$\partial _t u \;=\; \varvec{\text{ div }} \left( \frac{\varvec{\nabla }u}{1 + |\varvec{\nabla }u|^2/\lambda ^2}\right)$
Kornprobst et al. [29]	$$\partial _t u \;=\; {\left\{ \begin{array}{ll} \beta \Delta u &{} \text{ for } T < |\varvec{\nabla }u_\sigma |\\ \beta \Delta u - \gamma {{\,\textrm{sgn}\,}}(\partial _{\varvec{\eta }\varvec{\eta }} u_\sigma ) |\varvec{\nabla }u| &{} \text{ for } T \ge |\varvec{\nabla }u_\sigma |. \end{array}\right. }$$
Fu et al. [19]	$$\partial _t u \, = \, {\left\{ \begin{array}{ll} \frac{\partial _{\varvec{\xi \xi }} u}{1+\ell _1 \partial _{\varvec{\xi \xi }} u} - \textrm{sgn}(\partial _{\varvec{\eta \eta }} u_\sigma )|\varvec{\nabla }u| &{}\text{ for } |\varvec{\nabla }u_\sigma | >T_1\\ \Big ( \frac{\partial _{\varvec{\xi \xi }} u}{1+\ell _1 \partial _{\varvec{\xi \xi }} u} - |\textrm{th}(\ell _2 \partial _{\varvec{\eta }\varvec{\eta }} u) |\textrm{sgn}(\partial _{\varvec{\eta }\varvec{\eta }} u_\sigma )|\varvec{\nabla }u| \Big ) &{} \text{ for } |\varvec{\nabla }u_\sigma | \in (T_2, T_1]\\ \Delta u &{} \text{ else }. \end{array}\right. }$$
Total Variation [44]	$E(u) \;=\; \int \limits _{\Omega } \Big (\frac{1}{2}(u-f)^2 - \alpha |\varvec{\nabla }u|\Big ) d\varvec{x}$
Tschumperlé [47]	$\partial _t u = \text{ tr }(\varvec{T} \mathcal {H}) + \frac{2}{\pi } (\varvec{\nabla }u)^\top \int \limits ^\pi _{0} \varvec{\mathcal {{J}}}_{\sqrt{\varvec{T}}\varvec{a}_\phi } \sqrt{\varvec{T}} \varvec{a}_\phi d\phi$
Bornemann–März [5]	Algorithmic approach
EED [49]	$\partial _t u \;=\; \varvec{\text{ div }} \left( \varvec{D} \varvec{\nabla }u\right)$
Euler’s Elastica [32, 33]	$E(u) \;=\; \int \limits _{\Omega } |\varvec{\nabla }u|(b+(1-b)\kappa ^2 (u)) d\varvec{x}$

We use the following notations: $\varvec{D}$: Diffusion tensor, $\mathcal {H}$: Hessian, $\varvec{a}_\phi = (\cos \phi , \sin \phi )^\top$, $\varvec{\mathcal {{J}}}_{\sqrt{\varvec{T}}\varvec{a}_\phi }$: Jacobian of the vector field $\Omega \rightarrow \sqrt{\varvec{T}}\varvec{a}_\phi$, $\varvec{T}$: smoothing tensor, $\alpha , \beta , \gamma , \ell _1, \ell _2, b$: weights, $T_1, T_2, T$: thresholds, $\kappa$: curvature, *th*: $\tanh$, *tr*: trace

### Shape Completion

Shape completion is a special case of inpainting, in which data is given by a few parts of a shape. The goal is the reconstruction of the original shape. This is an especially difficult problem for many inpainting operators: It requires very high directional accuracy, the ability to bridge large gaps in the data and to create perfectly sharp edges. Let us now evaluate the performance of our RDS inpainting in the task of shape completion.

**Fig. 6:**
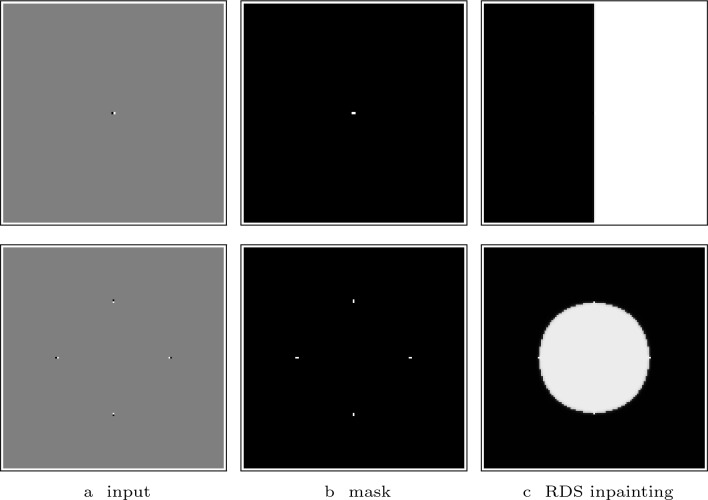
RDS inpainting from dipoles. **Top**: $128\times 128$ image; $\sigma = 2$, $\lambda = 1$. **Bottom**: $127\times 127$ image; $\sigma = 1.8$, $\lambda = 3.2$

Figure 6 shows two challenging examples. Here the goal is to reconstruct a half-plane from only one dipole (i.e. a white pixel next to a black one), and a disk from four dipoles. In both cases, RDS inpainting shows a flawless performance and recovers the desired shapes with the desired sharpness.

**Fig. 7:**
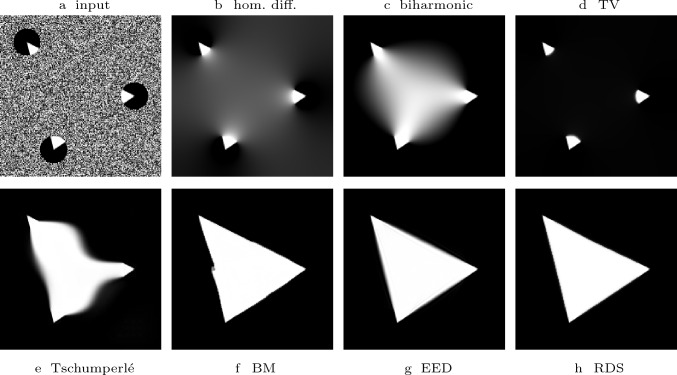
Comparison of inpainting methods. **Top**: Input image with known data in the disks and noise in the unknown region, homogeneous diffusion, biharmonic interpolation, and TV inpainting. **Bottom**: Tschumperlé’s approach, Bornemann–März (BM) method, EED inpainting, RDS inpainting with $\sigma = 3.5$ and $\lambda = 3$. All images apart from (h) were provided to us by the authors of [42]

To evaluate the performance of RDS inpaiting in comparison to various other PDE-based inpainting techniques, we extend an experiment performed by Schmaltz et al. [42] in Fig. 7. Inspired by the Kanisza triangle, the goal is the reconstruction of a white triangle on a black background of the data given in the disks. Table 1 shows the energies / evolution equations associated with each method. Clearly, homogeneous diffusion [8] and biharmonic interpolation [14], create a very blurry result which is typical for these linear methods. Total variation (TV) inpainting [44] fills the whole area in black. The directional artefacts created by the method of Tschumperlé [47] hint at suboptimal numerics in the original paper. Due to its suitability for connecting level lines, the Bornemann–März model [5] creates a satisfactory result but suffers from directional inaccuracies. Edge-enhancing diffusion (EED) [42, 55] reconstructs a perfect triangle. Schmaltz et al. [42] attribute this high performance to the anisotropy and the semilocality of the approach. By semilocality they refer to the fact, that EED uses neighbourhood information rather than acting purely local. RDS inpainting shares these properties. The coherence-enhancing shock term introduces a strong anisotropy, and the presmoothing procedures create semilocality. It also creates a high quality result without any directional artefacts, and the created edges are even sharper than those created by EED. Additionally, in contrast to EED our numerical algorithm for RDS inpainting also provides a maximum–minimum principle in the discrete case.

**Fig. 8:**
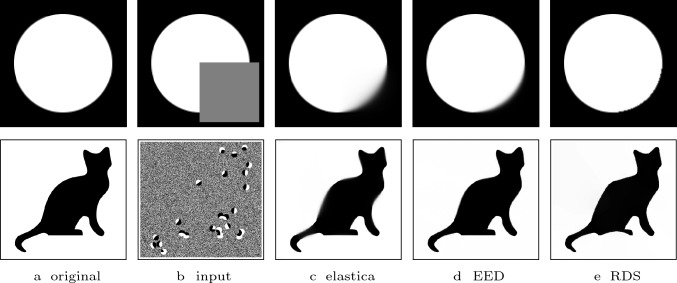
Comparison of Euler’s elastica, EED, and RDS inpainting. Parameters for RDS inpainting: **Top**: $\sigma = 2.5$ and $\lambda = 2$. **Bottom:** $\sigma = 2.1$ and $\lambda = 5.5$

In Fig. 8, we compare the performance of RDS inpainting to EED [49] and Euler’s elastica [32, 33], two methods that produce state-of-the-art results in the context of shape completion. Table 1 shows the evolution equation of EED and the energy functional that corresponds to Euler’s elastica. The results of Euler’s elastica are published in [43] and were given to us by the authors. The cat data and the EED inpainting of the cat are the original images from [53] that were provided to us by the author. In both examples RDS inpainting shows similar results as EED and Euler’s elastica. Overall, RDS inpainting creates the sharpest results.

### Evaluation of the Guidance Function

**Fig. 9:**
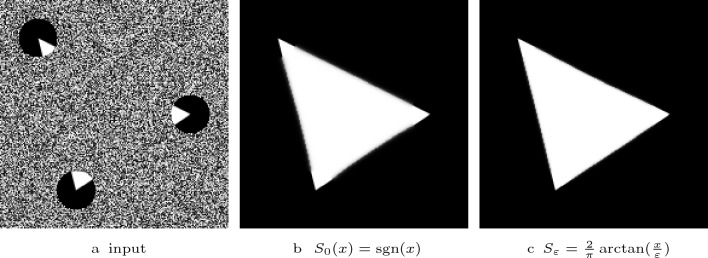
Comparison of diffusion–shock inpainting with parameter coupling guided by **a** ${{\,\textrm{sgn}\,}}$ and $\arctan$ function. Parameters: **b** $\sigma =5.8$, $\nu =\rho =1.8\, \sigma$, $\lambda = 3.5$, and **c** $\sigma = 3.5$, $\lambda = 3$

In the model of RDS inpainting, we use an $\arctan$ function as the guidance function, whereas the original diffusion-shock inpainting from [41] relies on the ${{\,\textrm{sgn}\,}}$ function. While the original model has already provided high quality results, it requires the optimisation of four parameters, which makes the method difficult to use in practice. In order to address this drawback, we have proposed a parameter coupling in a previous section. Applying these ideas to diffusion–shock inpainting based on a ${{\,\textrm{sgn}\,}}$ function decreases the inpainting quality. This is not the case for RDS inpainting. Figure 9 demonstrates this by the triangle reconstruction example. Clearly, the $\arctan$-guided result is superior: It creates sharper edges and reproduces a better directional accuracy.

**Fig. 10:**
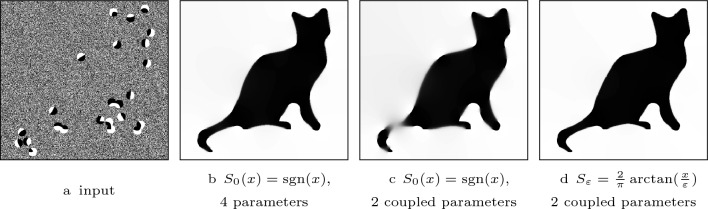
Comparison of diffusion–shock inpainting with parameter coupling guided by **a** ${{\,\textrm{sgn}\,}}$ and $\arctan$ function, and the result from our conference publication. Parameters: **b** $\sigma = 4.2$, $\rho =4.8$, $\nu =4.5$, and $\lambda = 7$, **c** $\sigma = 4.3$, $\lambda = 5.4$, $m= 1.8$ and **d** $\sigma = 2.1$ and $\lambda = 5.5$

**Fig. 11:**
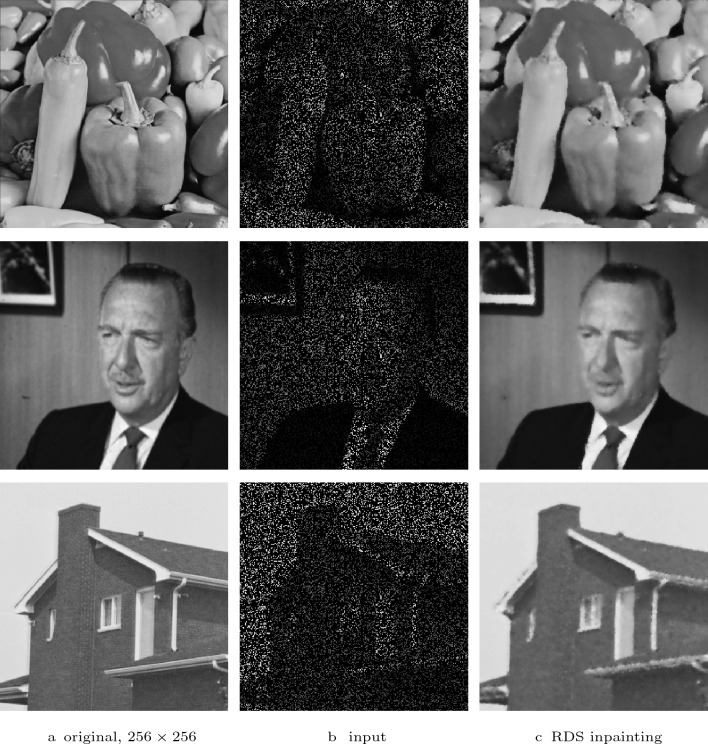
RDS inpainting of sparse greyscale images (20% randomly selected pixels). **First row**: $\sigma = 1.5$, $\lambda = 5$. **Second row**: $\sigma = 2.1$, $\lambda = 4$. **Third row**: $\sigma = 2.1$, $\lambda = 4.5$

**Fig. 12:**
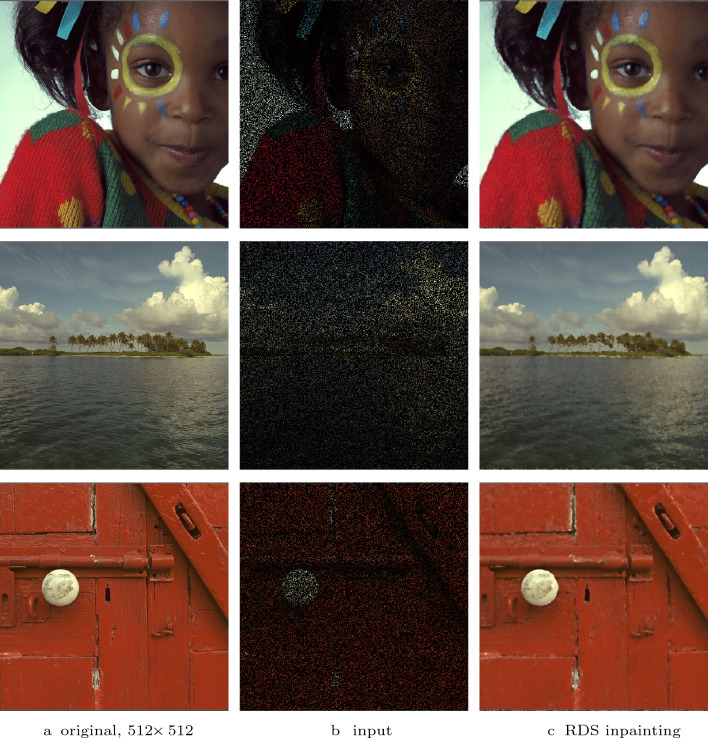
RDS inpainting for sparse colour images (20% randomly selected pixels). **First row**: $\sigma = 3$, $\lambda = 3$. **Second row**: $\sigma = 1.5$, $\lambda = 5$. **Third row**: and $\sigma = 1.5$, $\lambda = 4$

The cat reconstruction experiment in Fig. 10 makes this even more apparent. Here, we compare the result from Fig. 5 of our conference publication [41] which used a ${{\,\textrm{sgn}\,}}$-guided diffusion-shock inpainting without parameter coupling (b), ${{\,\textrm{sgn}\,}}$-guided diffusion-shock inpainting with parameter coupling (c) and RDS inpainting with parameter coupling (d). Clearly, RDS inpainting with parameter coupling creates a result that is very similar to the conference publication. However, the ${{\,\textrm{sgn}\,}}$-guided diffusion-shock inpainting is not able reconstruct the cat in a satisfactory way. This highlights the necessity of the regularisation in RDS inpainting for a parameter coupling that does not lead to a loss of inpainting quality.

### Natural Images

So far, we considered only binary images since they are especially challenging for PDE-based inpainting techniques. In Figs. 11 and  12, we show that RDS inpainting is also a suitable method for the reconstruction of natural images from sparse data. Figure 11 shows this for greyscale images of size $256 \times 256$. There, the runtime was 2.3 s for the *peppers* image, 2.5 s for the *walter* image and 2 s for the *house* image on a PC with an Intel^©^ Core™i9-11900K CPU @ 3.50 GHz.

Figure 12 depicts several RDS inpainting results created from sparse colour images. The original images are cropped versions of images from the Kodak dataset [16]. The sparse data are created by randomly selecting 20% of the pixels. The results show the effect of using a joint structure tensor and a joint weighting function for vector-valued images: Edges are formed in a synchronised way, and no unexpected colours or colour artefacts are introduced.

## Conclusions and Future Work

We have proposed regularised diffusion–shock (RDS) inpainting as an extension of our diffusion–shock inpainting from [41]. Diffusion–shock inpainting is the first method to utilise the perfect sharpness and directional accuracy of a coherence-enhancing shock filter [52] in the field of inpainting. Together with homogeneous diffusion [26, 27, 54], it creates results that rival the quality of popular PDE-based inpainting operators such as edge-enhancing diffusion [49] and Euler’s elastica [32, 33]. However, in contrast to these methods, its numerical algorithm also satisfies a maximum–minimum principle in the discrete case.

RDS inpainting introduces a regularisation to the original model. It stabilises the model w.r.t. the choice of parameters, and thereby allows the reduction of the number of parameters to two. This solves the largest disadvantage of the original diffusion–shock inpainting model from [41].

RDS inpainting is a second order integrodifferential process consisting of two simple components: homogeneous diffusion and coherence-enhancing shock filtering. We showed that it can offer equal or higher quality than higher order methods. However, higher order methods are algorithmically more challenging and often do not provide stability guarantees. On the other hand, our RDS inpainting allows a simple discretisation with an explicit scheme that provides a maximum–minimum principle. It constitutes a high quality second order integrodifferential process that questions the necessity of higher order methods in practice. This highlights the potential behind this class of methods, and we are aiming at gaining a deeper understanding of such integrodifferential processes in our ongoing work.

Most PDEs for inpainting are elliptic or parabolic. However, our results emphasise that hyperbolic processes deserve far more attention. They are a natural concept for modelling discontinuities, and shock filters are a prototype for this. For our application the coherence-enhancing shock filter in combination with homogeneous diffusion is the ideal choice. Interestingly, both components have been around for at least 20 years. This indicates that there still lies a huge potential in PDE-based inpainting, especially in hyperbolic concepts. Thus, we aim at exploring them further in our future work.

## Footnotes

1 Throughout our paper vectors are denoted by lower case boldface letters and matrices by upper case boldface letters.
